# Bayesian geostatistical modelling of soil-transmitted helminth survey data in the People’s Republic of China

**DOI:** 10.1186/1756-3305-6-359

**Published:** 2013-12-18

**Authors:** Ying-Si Lai, Xiao-Nong Zhou, Jürg Utzinger, Penelope Vounatsou

**Affiliations:** 1 Department of Epidemiology and Public Health, Swiss Tropical and Public Health Institute, P.O. Box, CH-4002 Basel, Switzerland; 2 University of Basel, Petersplatz 1, CH-4003 Basel, Switzerland; 3 National Institute of Parasitic Diseases, Chinese Center for Disease Control and Prevention, WHO Collaborating Centre for Malaria, Schistosomiasis and Filariasis, Key Laboratory of Parasite and Vector Biology, Ministry of Health, Shanghai 200025, People’s Republic of China

**Keywords:** Soil-transmitted helminths, *Ascaris lumbricoides*, *Trichuris trichiura*, Hookworm, Bayesian geostatistics, People’s Republic of China

## Abstract

**Background:**

Soil-transmitted helminth infections affect tens of millions of individuals in the People’s Republic of China (P.R. China). There is a need for high-resolution estimates of at-risk areas and number of people infected to enhance spatial targeting of control interventions. However, such information is not yet available for P.R. China.

**Methods:**

A geo-referenced database compiling surveys pertaining to soil-transmitted helminthiasis, carried out from 2000 onwards in P.R. China, was established. Bayesian geostatistical models relating the observed survey data with potential climatic, environmental and socioeconomic predictors were developed and used to predict at-risk areas at high spatial resolution. Predictors were extracted from remote sensing and other readily accessible open-source databases. Advanced Bayesian variable selection methods were employed to develop a parsimonious model.

**Results:**

Our results indicate that the prevalence of soil-transmitted helminth infections in P.R. China considerably decreased from 2005 onwards. Yet, some 144 million people were estimated to be infected in 2010. High prevalence (>20%) of the roundworm *Ascaris lumbricoides* infection was predicted for large areas of Guizhou province, the southern part of Hubei and Sichuan provinces, while the northern part and the south-eastern coastal-line areas of P.R. China had low prevalence (<5%). High infection prevalence (>20%) with hookworm was found in Hainan, the eastern part of Sichuan and the southern part of Yunnan provinces. High infection prevalence (>20%) with the whipworm *Trichuris trichiura* was found in a few small areas of south P.R. China. Very low prevalence (<0.1%) of hookworm and whipworm infections were predicted for the northern parts of P.R. China.

**Conclusions:**

We present the first model-based estimates for soil-transmitted helminth infections throughout P.R. China at high spatial resolution. Our prediction maps provide useful information for the spatial targeting of soil-transmitted helminthiasis control interventions and for long-term monitoring and surveillance in the frame of enhanced efforts to control and eliminate the public health burden of these parasitic worm infections.

## Background

Soil-transmitted helminths are a group of parasitic nematode worms causing human infection through contact with parasite eggs (*Ascaris lumbricoides* and *Trichuris trichiura*) or larvae (hookworm) that thrive in the warm and moist soil of the world’s tropical and subtropical countries [1]. More than 5 billion people are at risk of soil-transmitted helminthiasis [2]. Estimates published in 2003 suggest that 1,221 million people were infected with *A. lumbricoides*, 795 million with *T. trichiura* and 740 million with hookworms [3]. The greatest number of soil-transmitted helminth infections at that time occurred in the Americas, the People’s Republic of China (P.R. China), East Asia and sub-Saharan Africa [4]. Socioeconomic development and large-scale control efforts have lowered the number of people infected with soil-transmitted helminths in many parts of the world [1]. For the year 2010, the global burden due to soil-transmitted helminthiasis has been estimated at 5.2 million disability-adjusted life years [5].

In P.R. China, there have been two national surveys for parasitic diseases, including soil-transmitted helminthiasis. Both surveys used the Kato-Katz technique as the diagnostic approach, based on a single Kato-Katz thick smear obtained from one stool sample per individual. The first national survey was conducted from 1988 to 1992 and the second in 2001-2004. In the first survey, there were a total of 2,848 study sites with approximately 500 people examined per site. The survey indicated overall prevalences of 47.0%, 18.8% and 17.2% for *A. lumbricoides*, *T. trichiura* and hookworm infections, respectively, corresponding to 531 million, 212 million and 194 million infected people, respectively [6]. The second survey involved 687 study sites and there were 356,629 individuals examined overall. Analyses of the data revealed considerably lower prevalences for soil-transmitted helminth infections than in the first survey; *A. lumbricoides*, hookworm and *T. trichiura* prevalences were 12.7%, 6.1% and 4.6%, respectively [7]. However, interventions were less likely to reach marginalized communities in the poorest areas [8] and the diseases re-emerged whenever control measures were discontinued [9,10]. To overcome the challenge of parasite infections in P.R. China, in 2005, the Chinese Ministry of Health issued the “National Control Program on Important Parasitic Diseases from 2006 to 2015” with its target to reduce the prevalence of helminth infections by 70% by the year 2015 [8]. The key strategy for control was large-scale administration of anthelminthic drugs in high prevalence areas, especially targeting school-aged children and people living in rural areas [9,11].

Maps depicting the geographical distribution of the disease risk can aid control programmes to deliver cost-effective interventions and assist in monitoring and evaluation. The Coordinating Office of the National Survey on the Important Human Parasitic Diseases in P.R. China [7] obtained prevalence maps by averaging the data of the second national survey within each province. To our knowledge, high-resolution, model-based maps using available national survey data are not available to date in P.R. China. Model-based geostatistics predict the disease prevalence at places without observed data by quantifying the relation between the disease risk at observed locations with potential predictors such as socioeconomic, environmental, climatic and ecological information, the latter often obtained via remote sensing. Model-based geostatistics have been used before to map and predict the geographical distribution of soil-transmitted helminth infections in Africa [12,13], Asia and Latin America [14-16]. Model-based geostatistics typically employ regression analysis with random effects at the locations of the observed data. The random effects are assumed to be latent observations from a zero-mean Gaussian process, which models spatial correlation to the data via a spatially structured covariance. Bayesian formulations enable model fit via Markov chain Monte Carlo (MCMC) simulation algorithms [17,18] or other computational algorithms (e.g. integrated nested Laplace approximations (INLA) [19]). INLA is a computational approach for Bayesian inference and is an alternative to MCMC to overcome computational burden for obtaining the approximated posterior marginal distribution for the latent variables, as well as for the hyperparameters [20].

In this study, we aimed to: (i) identify the most important climatic, environmental and socioeconomic determinants of soil-transmitted helminth infections; and (ii) develop model-based Bayesian geostatistics to assess the geographical distribution and number of people infected with soil-transmitted helminths in P.R. China.

## Methods

### Ethical considerations

The work presented here is based on soil-transmitted helminth survey data derived from the second national survey and additional studies identified through an extensive review of the literature. All data in our study was extracted from published sources and they are aggregated over villages, towns or counties; therefore, do not contain information that is identifiable at individual or household level. Hence, there are no specific ethical considerations.

### Disease data

Geo-referenced data on soil-transmitted helminth infections from the second national survey conducted in P.R. China from 2001 to 2004 were provided by the National Institute of Parasitic Diseases, Chinese Center for Diseases Control and Prevention (IPD, China CDC; Shanghai, P.R. China). Moreover, an extensive literature search was undertaken in PubMed and China National Knowledge Internet (CNKI) from January 1, 2000 until April 25, 2013 to identify studies reporting village, town and county-level prevalence data of soil-transmitted helminth infections in P.R. China. Data were excluded if (i) they were from hospital surveys, post-intervention surveys, drug efficacy studies and clinical trials; (ii) reports on disease infection among travellers, military personnel, expatriates, mobile populations and other displaced or migrating populations; (iii) the geographical coordinates could not be identified; and (iv) the diagnostic technique was not reported [21]. Data were entered into the Global Neglected Tropical Diseases (GNTD) database, which is a geo-referenced, open-access source [21]. Geographical coordinates for the survey locations were obtained via Google maps, a free web mapping service application and technology system. As we focus on recent data pertaining to soil-transmitted helminth infections in P.R. China, we only considered surveys carried out from 2000 onwards.

### Climatic, demographic and environmental data

Climatic, demographic and environmental data were downloaded from different readily accessible remote sensing data sources, as shown in Table 1. Land surface temperature (LST) and normalized difference vegetation index (NDVI) were calculated to annual averages and land cover data was summarised to the most frequent category over the period of 2001-2004. Moreover, land cover data were re-grouped into six categories based on between-class similarities: (i) forest; (ii) shrubland and savanna; (iii) grassland; (iv) cropland; (v) urban; and (vi) wet areas. Monthly precipitation values were averaged to obtain a long-term average for the period 1950-2000. Four climatic zones were considered: (i) equatorial; (ii) arid; (iii) warm; and (iv) snow/polar. The following 13 soil types, which may be related to the viability of parasites or microorganisms living in the soil, were used: (i) percentage of coarse fragments (CFRAG, % >2 mm); (ii) percentage of sand (SDTO, mass %); (iii) percentage of silt (STPC, mass %); (iv) percentage of clay (CLPC, mass %); (v) bulk density (BULK, km/dm^3^); (vi) available water capacity (TAWC, cm/m); (vii) base saturation as percentage of ECEsoil (BSAT); (viii) pH measured in water (PHAQ); (ix) gypsum content (GYPS, g/kg); (x) organic carbon content (TOTC, g/kg); (xi) total nitrogen (TOTN, g/kg); (xii) FAO texture class (PSCL); and (xiii) FAO soil drainage class (DRAIN). Human influence index (HII) was included in the analysis to capture direct human influence on ecosystems [22]. Urban/rural extent was considered as a binary indicator. Gross domestic product (GDP) per capita was used as a proxy of people’s socioeconomic status. We obtained GDP per capita for each county from the P.R. China Yearbook full-text database in 2008.

**Table 1:** **Remote sensing data sources**^**a**^

**Source**	**Data type**	**Data period**	**Temporal resolution**	**Spatial resolution**
MODIS/Terra^b^	LST^j^	2001-2012	8 days	1 km
MODIS/Terra^b^	NDVI^k^	2001-2012	16 days	1 km
MODIS/Terra^b^	Land cover	2001-2004	Yearly	1 km
WorldClim^c^	Elevation	2000	-	1 km
WorldClim^c^	Precipitation	1950-2000	Monthly	1 km
SWBD^d^	Water bodies	2000	-	30 m
Köppen-Geiger^e^	Climate zones	1976-2000	-	50 km
ISRIC^f^	Soil types	-	-	8 km
Atlas of the biosphere^g^	Soil-moisture	1950-1999	-	50 km
SEDAC^h^	Population data	2000; 2010	-	5 km
SEDAC^h^	HII^l^	1995-2004	-	1 km
SEDAC^h^	Urban extents	1990-2000	-	1 km
China yearbook^i^	GDP per capita	2008	-	County-level

^a^Land cover data accessed on June 1, 2011; all other data accessed on April 1, 2013.

^b^Moderate Resolution Imaging Spectroradiometer (MODIS)/Terra; available at: https://lpdaac.usgs.gov/.

^c^Available at: http://www.worldclim.org/current.

^d^Shuttle Radar Topography Mission Water Body Data (SWBD); available at: http://gis.ess.washington.edu/data/vector/worldshore/index.html.

^e^World maps of Köppen-Geiger climate classification; available at: http://koeppen-geiger.vu-wien.ac.at/shifts.htm.

^f^International Soil Reference and Information Center; available at: http://www.isric.org/data/isric-wise-derived-soil-properties-5-5-arc-minutes-global-grid-version-12.

^g^Available at: http://www.sage.wisc.edu/atlas/maps.php?datasetid=23&includerelatedlinks=1&dataset=23.

^h^Socioeconomic Data and Applications Center;available at: http://sedac.ciesin.org/.

^i^China Yearbook full-text database; available at: http://acad.cnki.net/Kns55/brief/result.aspx?dbPrefix=CYFD.

^j^Land surface temperature (LST) day and night.

^k^Normalized difference vegetation index.

^l^Human influence index.

Moderate Resolution Imaging Spectroradiometer (MODIS) Reprojection Tool version 4.1 (EROS; Sioux Falls, USA) was applied to process MODIS/Terra data. All remotely sensed data were aligned over a prediction grid of 5 × 5 km spatial resolution using Visual Fortran version 6.0 (Digital Equipment Corporation; Maynard, USA). Data at the survey locations were also extracted in Visual Fortran. As the outcome of interest (i.e. infection prevalence with a specific soil-transmitted helminth species) is not available at the resolution of the covariates for surveys aggregated over counties, we linked the centroid of those counties with the average value of each covariate within the counties. Distances to the nearest water bodies were calculated using ArcGIS version 9.3 (ERSI; Redlands, USA). For county-level surveys, the distances of all the 5 × 5 km pixel centroids to their nearest water bodies within the county were extracted and averaged. The arithmetic mean was used as a summary measure of continuous data, while the most frequent category was used to summarise categorical variables.

### Statistical analysis

The survey year was grouped into two categories: before 2005 and from 2005 onwards. Land cover, climatic zones, soil texture and soil drainage were included into the model as categorical covariates. Continuous variables were standardised to mean 0 and standard deviation 1 using the command “std()” in Stata version 10 (Stata Corp. LP; College Station, USA). Pearson’s correlation was calculated between continuous variables. One of the two variables, which had correlation coefficient greater than 0.8, was dropped to avoid collinearity [23]. Preliminary analysis indicated that for this dataset, three categories were sufficient to encapsulate for non-linearity of continuous variables, therefore we constructed 3-level categorical variables based on their distribution. Subsequent variable selection incorporated within the geostatistical model selected the most probable functional form (linear *vs.* categorical). Bivariate and multivariate logistic regressions were carried out in Stata version 10.

Bayesian geostatistical logistic regression models with location-specific random effects were fitted to obtain spatially explicit soil-transmitted helminth infection estimates. Let *Y*_*i*_, *n*_*i*_ and *p*_*i*_ be the number of positive individuals, the number of those examined and the probability of infection at location *i* (*i* = 1, 2,…, *L*), respectively. We assume that *Y*_*i*_ arises from a binominal distribution *Y*_*i*_ *~ Bn*(*p*_*i*_,*n*_*i*_), where $\text{logit}\left(p_{i}\right)=\beta_{0}+\sum_{k=1}\beta_{k}\times X_{i}^{\left(k\right)}+\epsilon_{i}+\phi_{i}$. *β*_*k*_ is the regression coefficient of the *k*^*th*^ covariate $X_{i}^{\left(k\right)},$*ϵ*_*i*_ is a location-specific random effect and *ϕ*_*i*_ is an exchangeable non-spatial random effect. To estimate the parameters, we formulate our model in a Bayesian framework. We assumed ***ϵ*** = (*ϵ*_1_,…,*ϵ*_*L*_) followed a zero-mean multivariate normal distribution, ***ϵ*** *~ MVN*(0,Σ), where Matérn covariance function $\Sigma_{\mathrm{ij}}=\sigma_{\mathrm{sp}}^{2}{\left(\kappa d_{\mathrm{ij}}\right)}^{\upsilon }K_{\upsilon }\left(\kappa d_{\mathrm{ij}}\right)/\left(\Gamma \left(\upsilon \right)2^{\upsilon -1}\right).$*d*_*ij*_ is the Euclidean distance between locations *i* and *j. κ* is a scaling parameter, *υ* is a smoothing parameter fixed to 1 and *K*_*υ*_ denotes the modified Bessel function of second kind and order *υ*. The spatial range $\rho =\sqrt{8}/\kappa$, is the distance at which spatial correlation becomes negligible (<0.1) [24]. We assumed that *ϕ*_*i*_ follows a zero-mean normal distribution $\phi_{i}\sim N\left(0,\sigma_{\text{nonsp}}^{2}\right).$ A normal prior distribution was assigned to the regression coefficients, that is *β*_0_, *β*_*k*_ ∼ *N*(0, 1000) and loggamma priors were adopted for the precision parameters, $\tau_{\mathrm{sp}}=1/\sigma_{\mathrm{sp}}^{2}$ and $\tau_{\text{nonsp}}=1/\sigma_{\text{nonsp}}^{2}$ on the log scale, that is log(*τ*_*sp*_) ∼ log *gamma*(1, 0.00005) and log(*τ*_*nonsp*_) ∼ log *gamma*(1, 0.00005). Furthermore, we assumed the following prior distribution for range parameter log(*ρ*) ~ log *gamma*(1,0.01).

The most widely used computational approach for Bayesian geostatistical model fit is MCMC simulation. However, large spatial covariance matrix calculations can increase computational time and possibly introduce numerical errors. Hence, we fitted the geostatistical model using the stochastic partial differential equations (SPDE)/INLA [19,25] approach, readily implemented in the INLA R-package (available at: http://www.r-inla.org). Briefly, the spatial process assuming a Matérn covariance matrix Σ can be represented as a Gaussian Markov random field (GMRF) with mean zero and a symmetric positive definite precision matrix *Q* (defined as the inverse of Σ) [20]. The SPDE approach constructs a GMRF representation of the Matérn field on a triangulation (a set of non-intersecting triangles where any two triangles meet in at most a common edge or corner) partitioning the domain of the study region [25]. Subsequently, the INLA algorithm is used to estimate the posterior marginal (or joint) distribution of the latent Gaussian process and hyperparameters by Laplace approximation [19].

Bayesian variable selection, using normal mixture of inverse Gammas with parameter expansion (peNMIG) spike-and-slab priors [26] was applied on the model with independent random effect for each location to identify the best set of predictors (i.e. climatic, environmental and socioeconomic). In particular, we assumed a normal distribution for the regression coefficients with a hyperparameter for the variance *σ*_*B*_^2^ to be a mixture of inverse Gamma distributions, that is *β*_*k*_ ~ *N*(0,*σ*_*B*_^2^) where *σ*_*B*_^2^ ~ *I*_*k*_*IG*(*a*_*σ*_, *b*_*σ*_) + (1 - *I*_*k*_)*υ*_0_*IG*(*a*_*σ*_, *b*_*σ*_) and *a*_*σ*_*b*_*σ*_ are fixed parameters. *υ*_0_ is some small positive constant [27] and the indicator *I*_*k*_ has a Bernoulli prior distribution *I*_*k*_ ~ *bern*(*π*_*k*_), where *π*_*k*_ *~ beta*(*a*_*π*_,*b*_*π*_). We set (*a*_*σ*_,*b*_*σ*_) = (5,25) (*a*_*π*_,*b*_π_) = (1,1) and *υ*_0_ = 0.00025. The above prior of mixed inverse Gamma distributions is called a mixed spike and slab prior for *β*_*k*_ as one component of the mixture *υ*_0_*IG*(*a*_*σ*_,*b*_*σ*_) (when *I*_*k*_ = 0) is a narrow spike around zero that strongly shrinks *β*_*k*_ to zero, while the other component *IG*(*a*_*σ*_,*b*_*σ*_) (when *I*_*k*_ = 1) is a wide slab that moves *β*_*k*_ away from zero. The posterior distribution of *I*_*k*_ determines which component of the mixture is predominant contributing to the inclusion or exclusion of *β*_*k*_. For categorical variables, we applied a peNMIG prior developed by Scheipl *et al*. [26], which allows to include or exclude blocks of coefficients by improving “shrinkage” properties. Let *β*_*kh*_ be the regression coefficient for the *h*^*th*^ category of the *k*^*th*^ predictor, then *β*_*kh*_ = *a*_*k*_*ξ*_*hk*_, where *a*_*k*_ is assigned a NMIG prior described above and *ξ*_*hk*_ *~ N*(*m*_*hk*_,1). Here *m*_*hk*_ = *o*_*hk*_-(1-*o*_*hk*_) and *o*_*hk*_ ~ *bern*(0.5), allow to shrink |*ξ*_*hk*_| towards 1. Hence, *a*_*k*_ models the overall contribution of the *k*^*th*^ predictor and *ξ*_*hk*_ estimates the effects of each element *β*_*kh*_ of the predictor [27]. In addition, we introduced another indicator *I*_*d*_ for selection of either a categorical or a linear form of a continuous variable. Let *β*_*kd*1_ and *β*_*kd*2_ indicate coefficients of the categorical and linear form of *k*^*th*^ predictor, respectively, then *β*_*k*_ = *I*_*d*_*β*_*kd*1_ + (1 - *I*_*d*_)*β*_*kd*2_, where *I*_*d*_ ~ *Be*(0.5). MCMC simulation was employed to estimate the model parameters for variable selection in OpenBUGS version 3.0.2 (Imperial College and Medical Research Council; London, UK) [28]. Convergence was assessed by the Gelman and Rubin diagnostics [29], using the coda library in R [30]. In Bayesian variable selection, all models arising from any combination of covariates are fitted and the posterior probability for each model to be the true one is calculated. The predictors corresponding to the highest joint posterior probability of indicators (*I*_1_,*I*_2_,…*I*_*k*_,…,*I*_*K*_) were subsequently used as the best set of predictors to fit the final geostatistical model.

A 5 × 5 km grid was overlaid to the P.R. China map, resulting in 363,377 pixels. Predictions for each soil-transmitted helminth species were obtained via INLA at the centroids of the grid’s pixels. An overall soil-transmitted helminth prevalence was calculated assuming independence in the risk between any two species, that is, *p*_*S*_ = *p*_*A*_ + *p*_*T*_ + *p*_*h*_ - *p*_*A*_ × *p*_*T*_ - *p*_*A*_ × *p*_*h*_ - *p*_*T*_ × *p*_*h*_ + *p*_*A*_ × *p*_*T*_ × *p*_*h*_, where *p*_*S*_, *p*_*A*_, *p*_*T*_ and *p*_*h*_ indicate the predicted prevalence of overall soil-transmitted helminth, *A. lumbricoides*, *T. trichiura* and hookworm, respectively, for each pixel. The number of infected individuals at pixel level was estimated by multiplying the median of the corresponding posterior predictive distribution of the infection prevalence with the population density.

### Model validation

Our model was fitted on a subset of the data, including approximately 80% of survey locations. Validation was performed on the remaining 20% by estimating the mean predictive error (ME) between the observed *π*_*i*_ and predicted prevalence ${\hat{\pi }}_{i}$ at location *i*, where $\mathrm{ME}=1/N*\sum_{i=1}(\pi_{i}-{\hat{\pi }}_{i})$ and *N* is the total number of test locations. In addition, we calculated Bayesian credible intervals (BCI) of various probability and the percentages of observations included in these intervals.

## Results

### Data summaries

**Figure 1:**
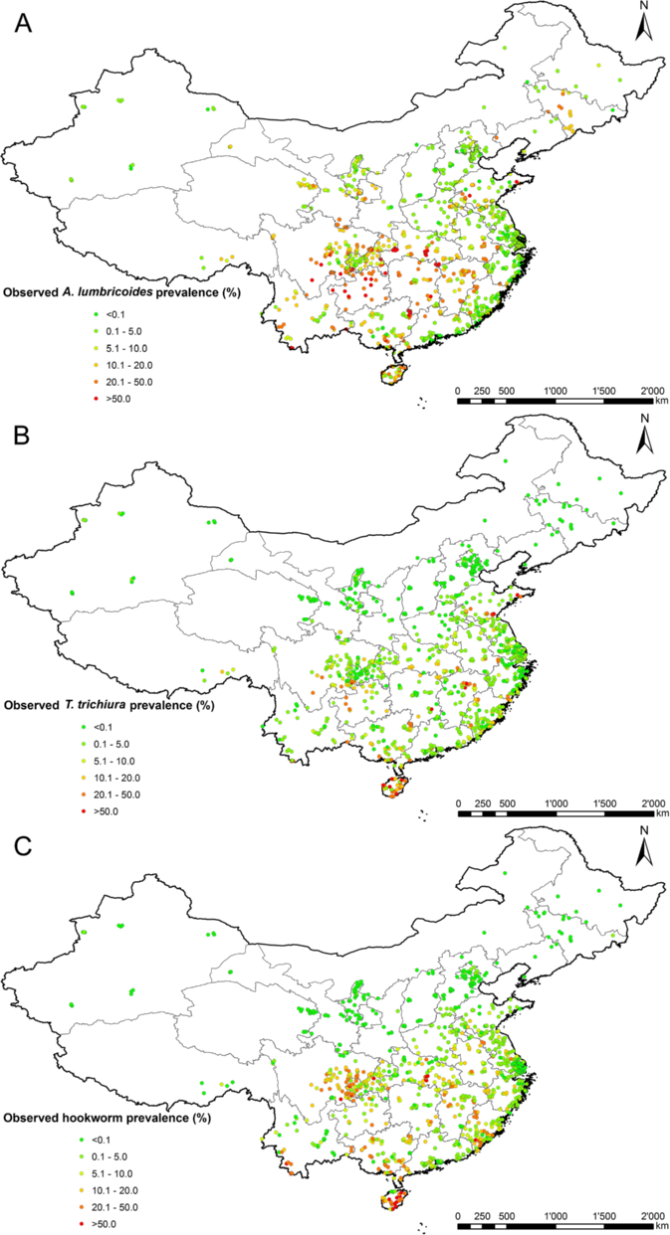
**Survey locations and observed prevalence across P.R. China.** The maps show the survey locations and observed prevalence for **(A) ***A. lumbricoides*, **(B) ***T. trichiura* and **(C)** hookworm.

The final dataset included 1,187 surveys for hookworm infection carried out at 1,067 unique locations; 1,157 surveys for *A. lumbricoides* infection at 1,052 unique locations; and 1,138 surveys for *T. trichiura* infection at 1,028 unique locations. The overall prevalence was 9.8%, 6.6% and 4.1% for *A. lumbricoides*, hookworm and *T. trichiura* infection, respectively. Details about the number of surveys by location type, study year, diagnostic method and infection prevalence are shown in Table 2. The geographical distribution of locations and observed prevalence for each soil-transmitted helminth species are shown in Figure 1. Maps of the spatial distribution of environmental/climatic, soil types and socioeconomic covariates used in Bayesian variable selection are provided in Additional file 1: Figure S1.

**Table 2:** Overview of the number of soil-transmitted helminth surveys

		***A. lumbricoides***	***T. trichiura***	**Hookworm**
		**Number of surveys (percentage)**		
Location types	Village/town	842 (72.8)	822 (72.2)	838 (70.6)
	County	315 (27.2)	316 (27.8)	349 (29.4)
Year of survey	2000-2004	739 (63.9)	737 (64.8)	775 (65.3)
	2005-2010	418 (36.1)	401 (35.2)	412 (34.7)
Diagnostic method	Kato-Katz	1124 (97.2)	1112 (97.7)	1151 (97.0)
	Stool sedimentation	3 (0.26)	3 (0.26)	3 (0.25)
	Flotation method^a^	16 (1.4)	16 (1.4)	19 (1.6)
	Ether-concentration^b^	1 (0.09)	1 (0.09)	1 (0.08)
	Other diagnostic method	13 (1.1)	6 (0.53)	13 (1.1)
Observed prevalence (%)	<0.1	126 (10.9)	374 (32.9)	364 (30.7)
	0.1-5.0	513 (44.3)	523 (46.0)	396 (33.4)
	5.1-10.0	152 (13.1)	95 (8.4)	149 (12.6)
	10.1-20.0	158 (13.7)	71 (6.2)	134 (11.3)
	20.1-50.0	155 (13.4)	58 (5.1)	118 (10.0)
	>50.0	53 (4.6)	17 (1.5)	26 (2.2)
Total		1,157 (100)	1,138 (100)	1,187 (100)

^a^Stool flotation method or McMaster salt flotation method.

^b^Formalin ethyl acetate concentration method.

### Spatial statistical modelling and variable selections

The models with the highest posterior probabilities selected the following covariates: GDP per capita, elevation, NDVI, LST at day, LST at night, precipitation, pH measured in water, and climatic zones for *T. trichiura*; GDP per capita, elevation, NDVI, LST at day, LST at night, precipitation, bulk density, gypsum content, organic carbon content, climatic zone and land cover for hookworm; and GDP per capita, elevation, NDVI, LST at day and climatic zone for *A. lumbricoides*. The corresponding posterior probabilities of the respective models were 33.2%, 23.6% and 21.4% for *T. trichiura*, hookworm and *A. lumbricoides*, respectively.

The parameter estimates that arose from the Bayesian geostatistical logistic regression fit are shown in Tables 3, 4 and 5. The infection risk of all three soil-transmitted helminth species decreased considerably from 2005 onwards. We found significant positive association between NDVI and the prevalence of *A. lumbricoides*. A negative association was found between GDP per capita, arid or snow/polar climatic zones and the prevalence of *A. lumbricoides*. High precipitation and LST at night are favourable conditions for the presence of hookworm, while high NDVI, LST at day, urban or wet land covers and arid or snow/polar climatic zones are less favourable. Elevation, LST at night, NDVI larger than 0.45 and equatorial climatic zone were associated with a higher odds of *T. trichiura* infection, while LST at day, arid or snow climatic zones were associated with a lower odds of *T. trichiura* infection.

**Table 3:** **Posterior summaries (median and 95% BCI) of the geostatistical model parameters for *A. lumbricoides***

	**Estimate**^†^
Year	0.34 (0.32; 0.36)^*^
GDP per capita (yuan)	
≤12,000	1.00
12,000-24,000	0.89 (0.68; 1.18)
>24,000	0.59 (0.41; 0.86)^*^
Elevation (m)	
≤55	1.00
55-400	1.21 (0.89; 1.63)
>400	1.54 (0.96; 2.46)
NDVI	
≤0.45	1.00
0.45-0.55	2.41 (2.05; 2.84)^*^
>0.55	1.30 (1.03; 1.64)^*^
LST at day (°C)	
≤21	1.00
21-23	1.07 (0.83; 1.37)
>23	1.08 (0.76; 1.54)
Climatic zone	
Warm	1.00
Equatorial	1.73 (0.42; 7.08)
Arid	0.41 (0.18; 0.98)^*^
Snow/polar	0.41 (0.20; 0.84)^*^
Range (km)	243.1 (182.8; 321.4)
Spatial variance (*σ*^2^_*sp*_)	2.64 (1.97; 3.51)
Non-spatial variance (*σ*^2^_*nonsp*_)	0.91 (0.77; 1.08)

^†^Regression coefficients are provided as odds ratios.

^*^Significant correlation based on 95% Bayesian credible interval (BCI).

**Table 4:** **Posterior summaries (median and 95% BCI) of the geostatistical model parameters for *T. trichiura***

	**Estimate**^†^
Year	0.26 (0.24; 0.28)^*^
GDP per capita	1.02 (0.83; 1.25)
Elevation	1.80 (1.37; 2.37)^*^
NDVI	
≤0.45	1.00
0.45-0.55	2.64 (1.99; 3.52)^*^
>0.55	1.59 (1.10; 2.32)^*^
LST at day	0.62 (0.48; 0.81)^*^
LST at night	3.61 (2.08; 6.32)^*^
Precipitation	1.23 (0.79; 1.91)
pH measured in water	
≤5.95	1.00
5.95-7.00	1.39 (1.00; 1.95)
>7.00	1.49 (0.96; 2.30)
Climatic zone	
Warm	1.00
Equatorial	6.40 (1.25; 31.49)^*^
Arid	0.10 (0.03; 0.36)^*^
Snow/polar	0.07 (0.02; 0.22)^*^
Range (km)	138.8 (104.3; 179.1)
Spatial variance (*σ*^2^_*sp*_)	4.19 (3.22; 5.08)
Non-spatial variance (*σ*^2^_*nonsp*_)	1.09 (0.88; 1.37)

^†^Regression coefficients are provided as odds ratios.

^*^Significant correlation based on 95% Bayesian credible interval (BCI).

**Table 5:** Posterior summaries (median and 95% BCI) of the geostatistical model parameters for hookworm

	**Estimate**^**†**^
Year	0.27 (0.25; 0.29)^*^
GDP per capita (yuan)	
≤12,000	1.00
12,000-24,000	1.28 (0.89; 1.85)
>24,000	0.89 (0.53; 1.50)
Elevation (m)	
≤55	1.00
55-400	1.34 (0.91; 1.98)
>400	1.32 (0.73; 2.38)
NDVI	
≤0.45	1.00
0.45-0.55	0.44 (0.36; 0.52)^*^
>0.55	0.36 (0.27; 0.47)^*^
LST at day	0.32 (0.23; 0.45)^*^
LST at night	7.35 (3.88; 14.12)^*^
Precipitation	3.17 (1.89; 5.48)^*^
Bulk density (km/dm^3^)	
≤1.29	1.00
1.29-1.36	0.82 (0.52; 1.30)
>1.36	0.66 (0.37; 1.17)
Gypsum content (g/kg)	
≤0	1.00
0-1	1.20 (0.88; 1.63)
>1	1.17 (0.73; 1.87)
Organic carbon content (g/kg)	
≤11	1.00
11-12.5	0.73 (0.44; 1.20)
>12.5	0.81 (0.46; 1.43)
Climatic zone	
Warm	1.00
Equatorial	1.87 (0.34; 10.13)
Arid	0.17 (0.03; 0.83)^*^
Snow/polar	0.05 (0.01; 0.21)^*^
Land cover	
Cropland	1.00
Forest	0.83 (0.57; 1.22)
Shrubland and savanna	1.07 (0.67; 1.70)
Grassland	0.63 (0.13; 2.58)
Urban	0.35 (0.22; 0.58)^*^
Wet areas	0.15 (0.07; 0.32)^*^
Range (km)	186.1 (126.8; 296.5)
Spatial variance (*σ*^2^_*sp*_)	5.07 (3.72; 6.63)
Non-spatial variance (*σ*^2^_*nonsp*_)	0.88 (0.69; 1.22)

^†^Regression coefficients are provided as odds ratios.

^*^Significant correlation based on 95% Bayesian credible interval (BCI).

### Model validation results

Model validation indicated that the Bayesian geostatistical logistic regression models were able to correctly estimate within a 95% BCI 84.2%, 81.5% and 79.3% for *T. trichiura*, hookworm and *A. lumbricoides*, respectively. A plot of coverage for the full range of credible intervals is presented in Additional file 2: Figure S2. The MEs for hookworm, *A. lumbricoides* and *T. trichiura* were 0.56%, 1.7%, and 2.0% respectively, suggesting that our model may slightly under-estimate the risk of each of the soil-transmitted helminth species.

### Predictive risk maps of soil-transmitted helminth infections

**Figure 2:**
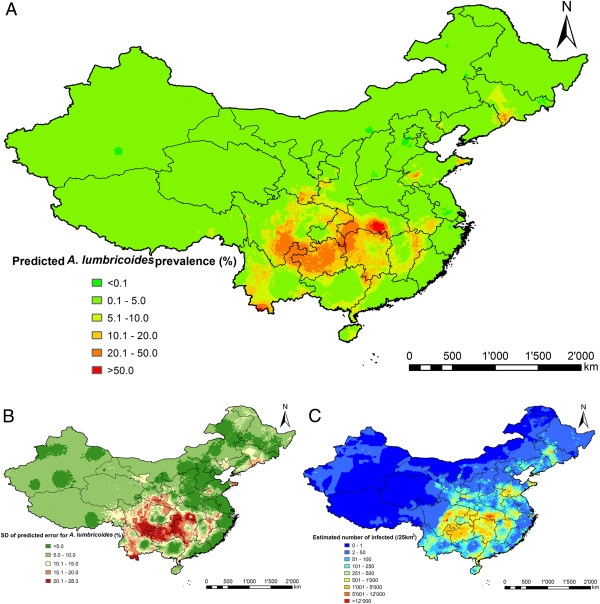
**The geographical distribution of *A. lumbricoides *infection risk in P.R. China.** The maps show the situation from 2005 onwards based on the median and standard deviation of the posterior predictive distribution. Estimates of **(A)** infection prevalence, **(B)** prediction uncertainty and **(C) ** number of infected individuals.

**Figure 3:**
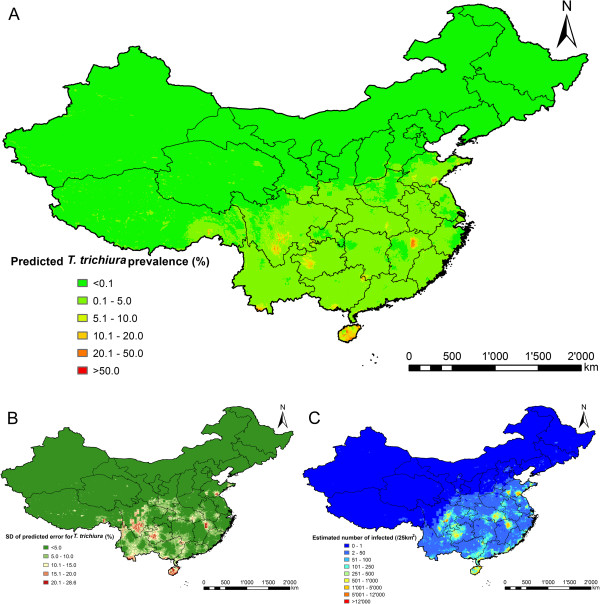
**The geographical distribution of *T. trichiura *infection risk in P.R. China.** The maps show the situation from 2005 onwards based on the median and standard deviation of the posterior predictive distribution. Estimates of **(A)** infection prevalence, **(B)** prediction uncertainty and **(C)** number of infected individuals.

**Figure 4:**
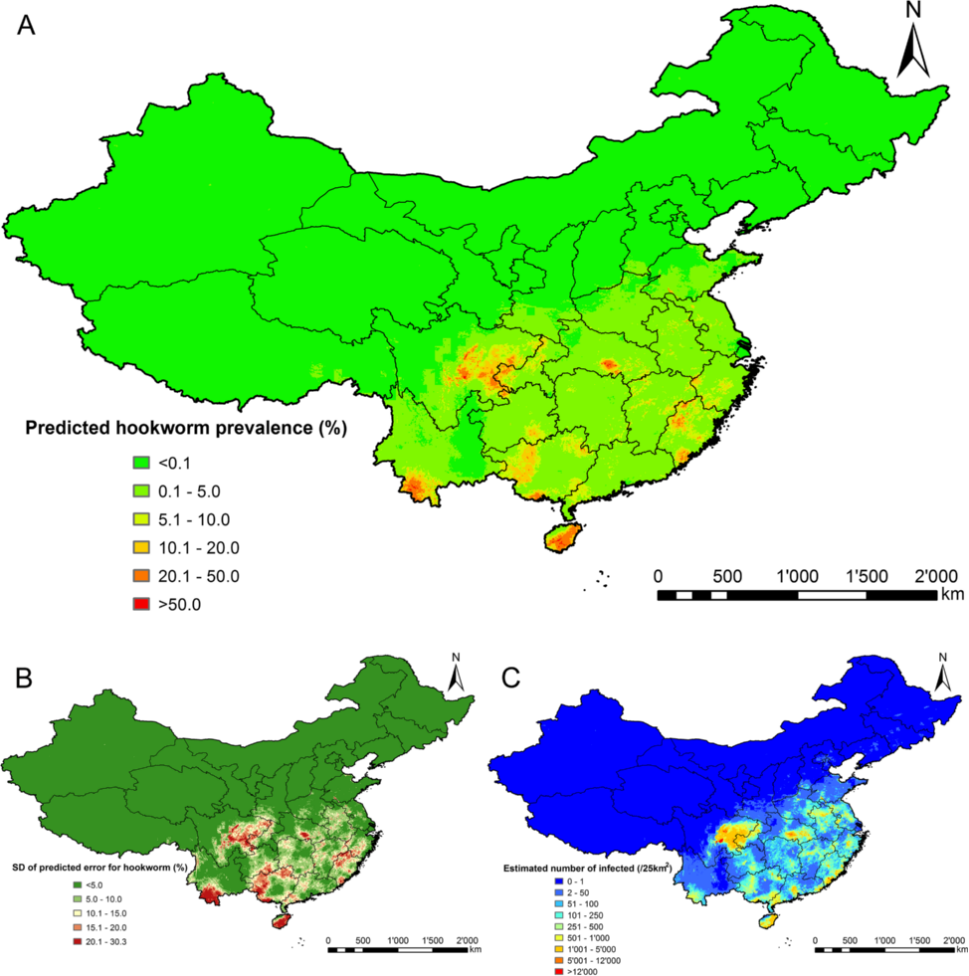
**The geographical distribution of hookworm infection risk in P.R. China.** The maps show the situation from 2005 onwards based on the median and standard deviation of the posterior predictive distribution. Estimates of **(A)** infection prevalence, **(B)** prediction uncertainty and **(C)** number of infected individuals.

Figures 2, 3 and 4 present species-specific predictive risk maps of soil-transmitted helminth infections for the period 2005 onwards. High prevalence of *A. lumbricoides* (>20%) was predicted in large areas of Guizhou province and the southern part of Sichuan and Hubei provinces. Moderate to high prevalence (5-20%) were predicted for large areas of Hunan, Yunnan, Jiangxi, some southern areas of Gansu and Anhui provinces and Chongqing city. For the northern part of P.R. China and the south-eastern coastal-line areas, low prevalences were predicted (<5%). The high prediction uncertainty shown in Figure 2B is correlated with high prevalence areas. High infection prevalence (>20%) with *T. trichiura* was predicted for a few small areas of the southern part of P.R. China. Moderate-to-high prevalence (5-20%) was predicted for large areas of Hainan province. High hookworm infection prevalence (>20%) was predicted for Hainan, eastern parts of Sichuan and southern parts of Yunnan provinces. Low prevalence (0.1-5%) of *T. trichiura* and hookworm infections were predicted for most areas of the southern part of P.R. China, while close to zero prevalence areas were predicted for the northern part.

### Estimates of number of people infected

**Figure 5:**
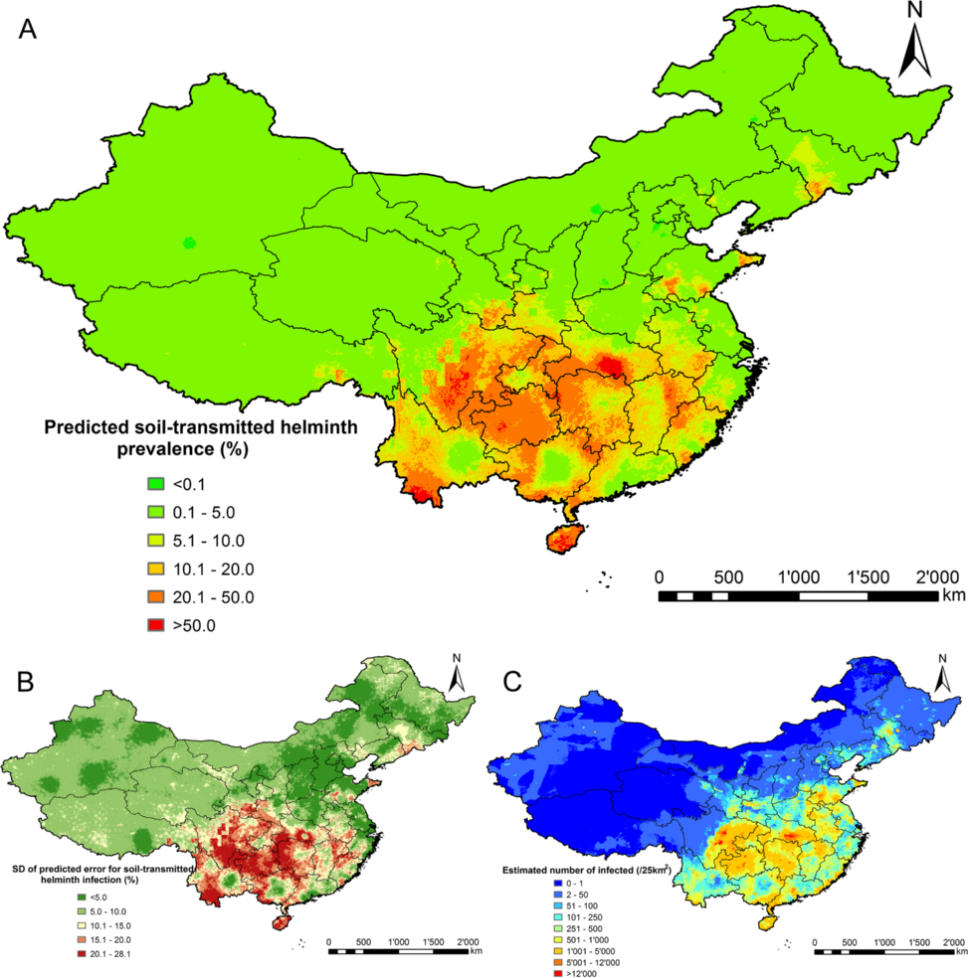
**The geographical distribution of soil-transmitted helminth infection risk in P.R. China. ** The maps show the situation from 2005 onwards based on the median and standard deviation of the posterior predictive distribution. Estimates of **(A)** infection prevalence, **(B)** prediction uncertainty and **(C)** number of infected individuals.

Figure 5 shows the combined soil-transmitted helminth prevalence and the number of infected individuals from 2005 onwards. Table 6 summarises the population-adjusted predicted prevalence and the number of infected individuals, stratified by province. The overall population-adjusted predicted prevalence of *A. lumbricoides*, hookworm and *T. trichiura* infections were, respectively, 6.8%, 3.7% and 1.8%, corresponding to 85.4, 46.6 and 22.1 million infected individuals. The overall population-adjusted predicted prevalence for combined soil-transmitted helminth infections was 11.4%.

**Table 6:** **Population-adjusted predicted prevalence (%) and number of individuals (×10**^**6**^**) infected with soil-transmitted helminths, stratified by province**^**†**^

**Province**	**Popula-tion**	***A. lumbricoides***		***T. trichiura***		**Hookworm**		**Any soil-transmitted helminth**	
		**Prevalence**	**No. of people infected**	**Prevalence**	**No. of people infected**	**Prevalence**	**No. of people infected**	**Prevalence**	**No. of people infected**
Anhui	54.9	4.3 (2.9; 6.8)	2.4 (1.6; 3.8)	1.4 (0.77; 2.6)	0.78 (0.42; 1.4)	4.6 (2.6; 8.1)	2.5 (1.5; 4.4)	10.1 (7.6; 14.2)	5.5 (4.2; 7.8)
Beijing	17.0	0.68 (0.40; 1.3)	0.12 (0.07; 0.22)	0.05 (0.02; 0.23)	0.01 (0.00; 0.04)	0.02 (0.01; 0.06)	0.00^#^ (0.00; 0.01)	0.77 (0.47; 1.4)	0.13 (0.08; 0.24)
Chongqing	26.7	10.2 (7.9; 12.4)	2.7 (2.1; 3.3)	1.2 (0.78; 1.7)	0.31 (0.21; 0.47)	10.3 (7.6; 13.5)	2.8 (2.0; 3.6)	20.6 (17.1; 24.0)	5.5 (4.6; 6.4)
Fujian	32.8	1.9 (1.5; 2.7)	0.63 (0.48; 0.89)	2.0 (1.4; 3.2)	0.67 (0.44; 1.0)	8.6 (6.4; 11.6)	2.8 (2.1; 3.8)	12.3 (10.1; 15.3)	4.0 (3.3; 5.0)
Gansu	25.6	6.6 (3.8; 11.5)	1.7 (0.97; 2.9)	0.30 (0.10; 1.3)	0.08 (0.02; 0.34)	0.02 (0.00; 0.16)	0.00^#^ (0.00; 0.04)	7.0 (4.1; 11.9)	1.8 (1.0; 3.0)
Guangdong	91.1	3.0 (2.1; 4.4)	2.7 (1.9; 4.0)	2.3 (1.4; 3.5)	2.1 (1.3; 3.2)	4.3 (2.6; 7.1)	3.9 (2.4; 6.5)	9.0 (7.1; 12.4)	8.2 (6.4; 11.3)
Guangxi	37.6	6.9 (5.1; 9.4)	2.6 (1.9; 3.5)	3.6 (2.3; 5.7)	1.4 (0.86; 2.1)	7.8 (5.4; 11.8)	2.9 (2.0; 4.4)	17.4 (14.0; 21.8)	6.5 (5.3; 8.2)
Guizhou	31.4	27.9^*^ (19.5; 37.6)	8.7 (6.1; 11.8)	5.2 (2.8; 9.6)	1.6 (0.87; 3.0)	4.1 (2.1; 7.7)	1.3 (0.65; 2.4)	34.6 (25.9; 43.3)	10.9 (8.1; 13.6)
Hainan	6.7	7.5 (5.0; 10.5)	0.50 (0.33; 0.70)	18.3^*^ (11.9; 25.9)	1.2 (0.80; 1.7)	22.1^*^ (16.0; 29.7)	1.5 (1.1; 2.0)	40.8^*^ (33.6; 48.6)	2.7 (2.2; 3.3)
Hebei	75.5	1.3 (0.77; 2.1)	0.95 (0.58; 1.6)	0.09 (0.04; 0.20)	0.07 (0.03; 0.15)	0.12 (0.06; 0.31)	0.09 (0.04; 0.23)	1.5 (0.97; 2.4)	1.1 (0.73; 1.8)
Heilongjiang	42.3	2.1 (0.99; 4.7)	0.90 (0.42; 2.0)	0.02 (0.01; 0.07)	0.01 (0.00; 0.03)	0.04 (0.01; 0.26)	0.02 (0.00; 0.11)	2.2 (1.1; 4.8)	0.93 (0.44; 2.0)
Henan	84.3	2.2 (1.5; 3.2)	1.8 (1.2; 2.7)	0.62 (0.34; 1.2)	0.52 (0.29; 1.1)	1.5 (0.83; 2.7)	1.2 (0.70; 2.3)	4.3 (3.2; 5.9)	3.6 (2.7; 4.9)
Hubei	58.2	18.3 (14.4; 22.7)	10.6 (8.4; 13.2)	3.2 (1.7; 6.7)	1.9 (0.98; 3.9)	5.9 (4.0; 8.9)	3.4 (2.3; 5.2)	24.9 (20.7; 30.1)	14.5 (12.0; 17.5)
Hunan	55.1	17.7 (12.5; 24.9)	9.7 (6.9; 13.7)	1.8 (0.9; 3.6)	0.99 (0.50; 2.0)	3.5 (2.0; 6.6)	1.9 (1.1; 3.7)	22.1 (16.6; 29.5)	12.2 (9.2; 16.3)
Jiangsu	74.3	1.2 (0.88; 1.8)	0.91 (0.65; 1.3)	0.72 (0.45; 1.5)	0.54 (0.34; 1.1)	2.2 (1.5; 3.5)	1.6 (1.1; 2.6)	4.1 (3.2; 5.7)	3.1 (2.4; 4.3)
Jiangxi	36.3	11.3 (8.1; 15.7)	4.1 (2.9; 5.7)	3.3 (2.0; 5.8)	1.2 (0.73; 2.1)	5.0 (3.1; 7.7)	1.8 (1.1; 2.8)	18.6 (14.8; 23.6)	6.8 (5.4; 8.6)
Jilin	29.2	7.2 (4.3; 11.8)	2.1 (1.2; 3.5)	0.02 (0.00; 0.09)	0.01 (0.00; 0.03)	0.04 (0.01; 0.28)	0.01 (0.00; 0.08)	7.3 (4.3; 11.9)	2.1 (1.3; 3.5)
Liaoning	43.1	3.5 (1.4; 9.1)	1.5 (0.61; 3.9)	0.02 (0.00; 0.08)	0.01 (0.00; 0.03)	0.03 (0.00; 0.21)	0.01 (0.00; 0.09)	3.5 (1.5; 9.2)	1.5 (0.64; 5.0)
Nei Mongol	29.7	2.2 (1.0; 5.2)	0.65 (0.30; 1.6)	0.01^#^ (0.01; 0.06)	0.00^#^ (0.00; 0.02)	0.01 (0.00; 0.04)	0.00^#^ (0.00; 0.01)	2.2 (1.0; 5.2)	0.66 (0.31; 1.6)
Ningxia Hui	6.3	3.8 (2.5; 5.4)	0.24 (0.16; 0.34)	0.07 (0.03; 0.26)	0.00^#^ (0.00; 0.02)	0.00^#^ (0.00; 0.03)	0.00^#^ (0.00; 0.00)	3.9 (2.6; 5.5)	0.25 (0.16; 0.34)
Qinghai	5.0	5.7 (3.4; 9.5)	0.28 (0.17; 0.47)	0.05 (0.01; 0.20)	0.00^#^ (0.00; 0.01)	0.00^#^ (0.00; 0.03)	0.00^#^ (0.00; 0.00)	5.8 (3.5; 9.6)	0.29 (0.17; 0.48)
Shaanxi	34.2	5.8 (2.6; 12.5)	2.0 (0.89; 4.3)	0.84 (0.30; 2.3)	0.29 (0.10; 0.78)	0.39 (0.09; 1.9)	0.14 (0.03; 0.66)	7.0 (3.7; 13.4)	2.4 (1.3; 4.6)
Shandong	93.4	5.2 (3.7; 7.3)	4.9 (3.4; 6.9)	2.1 (1.4; 3.3)	2.0 (1.3; 3.1)	0.78 (0.44; 1.6)	0.73 (0.41; 1.5)	7.9 (6.2; 10.4)	7.4 (5.8; 9.7)
Shanghai	15.0	0.32^#^ (0.21; 0.54)	0.05^#^ (0.03; 0.08)	0.46 (0.27; 0.81)	0.07 (0.04; 0.12)	0.02 (0.01; 0.05)	0.00^#^ (0.00; 0.01)	0.82 (0.58; 1.2)	0.12 (0.09; 0.18)
Shanxi	35.5	1.7 (0.88; 3.9)	0.59 (0.31; 1.4)	0.14 (0.04; 0.41)	0.05 (0.01; 0.15)	0.07 (0.02; 0.27)	0.02 (0.01; 0.10)	1.9 (1.1; 4.1)	0.68 (0.37; 1.4)
Sichuan	94.6	14.8 (11.5; 19.3)	14.0^*^ (10.9; 18.2)	3.9 (2.4; 6.9)	3.7^*^ (2.2; 6.5)	15.1 (10.9; 21.4)	14.3^*^ (10.3; 20.3)	30.6 (26.0; 36.2)	29.0^*^ (24.6; 34.2)
Tianjin	9.8	0.66 (0.32; 1.3)	0.06 (0.03; 0.13)	0.01^#^ (0.00; 0.05)	0.00^#^ (0.00; 0.00)	0.03 (0.01; 0.13)	0.00^#^ (0.00; 0.01)	0.70^#^ (0.36; 1.3)	0.07^#^ (0.04; 0.13)
Xinjiang Uygur	25.0	2.4 (1.1; 7.1)	0.60 (0.26; 1.8)	0.05 (0.02; 0.15)	0.01 (0.00; 0.04)	0.01 (0.00; 0.09)	0.00^#^ (0.00; 0.02)	2.5 (1.1; 7.2)	0.62 (0.28; 1.8)
Tibet	2.7	3.3 (1.5; 7.7)	0.09 (0.04; 0.21)	0.47 (0.15; 1.4)	0.01 (0.00; 0.04)	0.03 (0.01; 0.17)	0.00^#^ (0.00; 0.00)	3.9 (1.9; 8.1)	0.10 (0.05; 0.22)
Yunnan	39.5	13.6 (9.0; 19.4)	5.4 (3.5; 7.7)	3.5 (2.0; 6.5)	1.4 (0.77; 2.6)	2.7 (1.6; 5.0)	1.1 (0.62; 2.0)	19.0 (13.89; 25.1)	7.5 (5.5; 9.9)
Zhejiang	45.4	0.80 (0.58; 1.2)	0.36 (0.26; 0.54)	0.64 (0.38; 1.1)	0.29 (0.17; 0.50)	3.0 (1.9; 4.9)	1.4 (0.88; 2.2)	4.4 (3.3; 6.4)	2.0 (1.5; 2.9)
**Total**	**1,257.9**	**6.8 (6.2; 7.5)**	**85.4 (77.8; 94.0)**	**1.8 (1.5; 2.1)**	**22.1 (18.7; 26.2)**	**3.7 (3.2; 4.3)**	**46.6 (40.7; 53.7)**	**11.4 (10.8; 12.2)**	**143.8 (135.9; 153.8)**

^†^Estimates based on Gridded population of 2010; calculations based on the median and 95% BIC of the posterior distribution of the predicted risk from 2005 onwards.

*Highest prevalence/largest number of infected individuals among provinces; ^#^lowest prevalence/smallest number of infected individuals among provinces.

For *A. lumbricoides*, the predicted prevalence ranged from 0.32% (Shanghai) to 27.9% (Guizhou province). Shanghai had the smallest (0.05 million) and Sichuan province the largest number (14.8 million) of infected individuals. For *T. trichiura*, the predicted prevalence ranged from 0.01% (Tianjin) to 18.3% (Hainan province). The smallest number of infected individuals were found in Nei Mongol, Ningxia Hui, Qinghai provinces and Tianjin (<0.01 million) whereas the largest number, 3.7 million, was predicted for Sichuan province. For hookworm, Ningxia Hui and Qinghai province had the lowest predicted prevalence (<0.01%), while Hainan province had the highest (22.1%). The provinces of Gansu, Nei Mongol, Ningxia Hui, Qinghai, Xinjiang Uygur and Tibet, and the cities of Beijing, Shanghai and Tianjin each had less than 10,000 individuals infected with hookworm. Sichuan province had the largest predicted number of hookworm infections (14.3 million).

The predicted combined soil-transmitted helminth prevalence ranged from 0.70% (Tianjin) to 40.8% (Hainan province). The number of individuals infected with soil-transmitted helminths ranged from 0.07 million (Tianjin) to 29.0 million (Sichuan province). Overall, slightly more than one out of ten people in P.R. China is infected with soil-transmitted helminths, corresponding to more than 140 million infections in the year 2010.

## Discussion

To our knowledge, we present the first model-based, nation-wide predictive infection risk maps of soil-transmitted helminths for P.R. China. Previous epidemiological studies [7] were mainly descriptive, reporting prevalence estimates at specific locations or visualized at province level using interpolated risk surface maps. We carried out an extensive literature search and collected published georeferenced soil-transmitted helminth prevalence data across P.R. China, alongside the ones from the second national survey that had been completed in 2004. Bayesian geostatistical models were utilised to identify climatic/environmental and socioeconomic factors that were significantly associated with infection risk, and hence, the number of infected individuals could be calculated at high spatial resolution. We derived species-specific risk maps. Additionally, we produced a risk map with any soil-transmitted helminth infection, which is particularly important for the control of soil-transmitted helminthiasis, as the same drugs (mainly albendazole and mebendazole) are used against all three species [31,32].

Model validation suggested good predictive ability of our final models. In particular, 84.2%, 81.5% and 79.3% of survey locations were correctly predicted within a 95% BCI for *T. trichiura*, hookworm and *A. lumbricoides*, respectively. The combined soil-transmitted helminth prevalence (11.4%) is supported by the current surveillance data reported to China CDC that shows infection rates in many areas of P.R. China around 10%. We found that all ME were above zero, hence the predictive prevalence slightly under-estimated the true prevalence of each of the three soil-transmitted helminth species. The combined soil-transmitted helminth prevalence estimates assume that the infection of each species is independent of each other. However, previous research reported significant associations, particularly between *A. lumbricoides* and *T. trichiura*[33,34]. Hence, our assumption may over-estimate the true prevalence of soil-transmitted helminths. Unfortunately we do not have co-infection data from P.R. China, and thus we are unable to calculate a correction factor.

Our results indicate that several environmental and climatic predictors are significantly associated with soil-transmitted helminth infections. For example, LST at night was significantly associated with *T. trichiura* and hookworm, suggesting that temperature is an important driver of transmission. Similar results have been reported by other researchers [2,35]. Our results suggest that the risk of infection with any of the soil-tansmitted helminth species is higher in equatorial or warm zones, compared to the arid and snow/polar zones. This is consistent with earlier findings that extremely arid environments limit the transmission of soil-transmitted helminths [2], while equatorial or warm zones provide temperatures and soil moisture that are particularly suitable for larval development [35]. However, we found a positive association between elevation and *T. trichiura* infection risk, which contradicts earlier reports [36,37]. The reason may be the altitude effect, i.e. the negative correlation between altitude and economy in P.R. China [38]. The low socioeconomic development in high altitude or mountainous areas might result in limited access to healthcare services [39,40].

On the other hand, it is reported that socioeconomic factors are closely related with the behaviour of people, which in turn impacts the transmission of soil-transmitted helminths [41]. Indeed, wealth, inadequate sewage discharge, drinking of unsafe water, lack of sanitary infrastructure, personal hygiene habits, recent travel history, low education and demographic factors are strongly associated with soil-transmitted helminth infections [42-46]. Our results show that GDP per capita has a negative effect on *A. lumbricoides* infection risk. Other socioeconomic proxies such as sanitation level, number of hospital beds and percentage of people with access to tap water might be more readily able to explain the spatial distribution of infection risk.

Model-based estimates adjusted for population density indicate that the highest prevalence of *A. lumbricoides* occurred in Guizhou province. *T. trichiura* and hookworm were most prevalent in Hainan province. Although the overall soil-transmitted helminth infection risk decreased over the past several years, Hainan province had the highest risk in 2010, followed by Guizhou and Sichuan provinces. These results are consistent with the reported data of the second national survey on important parasitic diseases [7], and hence more effective control strategies are needed in these provinces.

The targets set out by the Chinese Ministry of Health in the “National Control Program on Important Parasitic Diseases from 2006 to 2015” are to reduce the prevalence of soil-transmitted helminth infections by 40% until 2010 and up to 70% until 2015 [8]. The government aims to reach these targets by a series of control strategies, including anthelminthic treatment, improvement of sanitation, and better information, education and communication (IEC) campaigns [47]. Preventive chemotherapy is recommended for populations older than 3 years in areas where the prevalence of soil-transmitted helminth infection exceeds 50%, while targeted drug treatment is recommended for children and rural population in areas where infection prevalences range between 10% and 50% [48]. Our models indicate that the first step of the target, i.e. reduction of prevalence by 40% until 2010, has been achieved. Indeed, the prevalence of *T. trichiura*, hookworm and *A. lumbricoides* dropped from 4.6%, 6.1% and 12.7% in the second national survey between 2001 and 2004 [7] to 1.8%, 3.7% and 6.8% in 2010, which corresponds to respective reductions of 60.9%, 39.3% and 46.5%. The combined soil-transmitted helminth prevalence dropped from 19.6% to 11.4% in 2010, a reduction of 41.8%. These results also suggest that, compared to *T. trichiura* and *A. lumbricoides*, more effective strategies need to be tailored for hookworm infections.

The data of our study stem largely from community-based surveys. However, the information extracted from the literature is not disaggregated by age, and hence we were not able to obtain age-adjusted predictive risk maps. In addition, more than 96% of observed surveys used the Kato-Katz technique [49,50]. We assumed that the diagnostic sensitivity was similar across survey locations. However, the sensitivity depends on the intensity of infection, and hence varies in space [51]. The above data limitations are known in geostatistical meta-analyses of historical data [27] and we are currently developing methods to address them.

## Conclusion

The work presented here is the first major effort to present model-based estimates of the geographical distribution of soil-transmitted helminth infection risk across P.R. China, and to identify the associated climatic, environmental and socioeconomic risk factors. Our prediction maps provide useful information for identifying priority areas where interventions targeting soil-transmitted helminthiasis are most urgently required. In a next step, we plan to further develop our models to address data characteristics and improve model-based predictions.

## Abbreviations

BCI: Bayesian credible interval; BSAT: Base saturation as percentage of ECEsoil; BULK: Bulk density; CFRAG: Percentage of coarse fragments; China CDC: Chinese center for diseases control and prevention; CLPC: Percentage of clay; CNKI: China national knowledge internet; DRAIN: FAO soil drainage class; GDP: Gross domestic product; GMRF: Gaussian Markov random field; GNTD database: Global neglected tropical diseases database; GYPS: Gypsum content; HII: Human influence index; IEC: Information, education, and communication; INLA: Integrated nested Laplace approximations; IPD: National Institute of Parasitic Diseases; LST: Land surface temperature; MCMC: Markov chain Monte Carlo; MODIS: Moderate Resolution Imaging Spectroradiometer; NDVI: Normalized difference vegetation index; P.R. China: People’s Republic of China; peNMIG: Normal mixture of inverse Gammas with parameter expansion; PHAQ: pH measured in water; PSCL: FAO texture class; SPDE: Stochastic partial differential equations; TAWC: Available water capacity; TOTC: Organic carbon content; TOTN: Total nitrogen; SDTO: Percentage of sand; STPC: Percentage of silt.

## Competing interests

The authors have declared that no competing interests exist.

## Authors’ contributions

YSL and PV analyzed the data. YSL, JU and PV wrote the paper. PV, JU and XNZ conceptualized the project. XNZ provided data. YSL did the literature review and processed the data. PV, JU and XNZ provided important intellectual content. All authors read and approved the originally submitted and the revised manuscript.

## Supplementary Material

Additional file 1: Figure S1 Spatial distribution of environmental/climatic, soil types and socioeconomic factors in P.R. China. Click here for file

Additional file 2: Figure S2 Model validation results. Percentage of survey locations with observed prevalence included within the Bayesian credible interval (BCI) of various probability coverage cut-offs (bar plots) calculated from the posterior predicted distribution. Solid lines indicate the corresponding width of BCI. Click here for file

## References

[B1] Bethony J Brooker S Albonico M Geiger SM Loukas A Diemert D Hotez PJ Soil-transmitted helminth infections: ascariasis, trichuriasis, and hookworm Lancet 2006 6 1521 1532 10.1016/S0140-6736(06)68653-4 16679166

[B2] Pullan RL Brooker SJ The global limits and population at risk of soil-transmitted helminth infections in 2010 Parasit Vectors 2012 6 81 10.1186/1756-3305-5-81 22537799 PMC3419672

[B3] de Silva NR Brooker S Hotez PJ Montresor A Engels D Savioli L Soil-transmitted helminth infections: updating the global picture Trends Parasitol 2003 6 547 551 10.1016/j.pt.2003.10.002 14642761

[B4] Hotez PJ Bundy DAP Beegle K Brooker S Drake L de Silva N Montresor A Engels D Jukes M Chitsulo L Jamison DT, Breman JG, Measham AR, Alleyne G, Claeson M, Evans DB, Jha P, Mills A, Musgrove P Helminth infections: soil-transmitted helminth infections and schistosomiasis Disease Control Priorities in Developing Countries 2006 2 Washington, DC: World Bank 467 482 21250326

[B5] Murray CJL Vos T Lozano R Naghavi M Flaxman AD Michaud C Ezzati M Shibuya K Salomon JA Abdalla S Disability-adjusted life years (DALYs) for 291 diseases and injuries in 21 regions, 1990-2010: a systematic analysis for the global burden of disease study 2010 Lancet 2012 6 2197 2223 10.1016/S0140-6736(12)61689-4 23245608

[B6] Xu LQ Yu SH Jin ZX Yang JL Lai CQ Zhang XJ Zheng CQ Soil-transmitted helminthiases - nationwide survey in China Bull World Health Organ 1995 6 507 513 7554023 PMC2486772

[B7] Coordinating Office of the National Survey on the Important Human Parasitic Diseases A national survey on current status of the important parasitic diseases in human population Chin J Parasitol Parasit Dis 2005 6 332 340 (in Chinese) 16562464

[B8] Zheng Q Chen Y Zhang HB Chen JX Zhou XN The control of hookworm infection in China Parasit Vectors 2009 6 44 10.1186/1756-3305-2-44 19775473 PMC2760515

[B9] Li T He SY Zhao H Zhao GH Zhu XQ Major trends in human parasitic diseases in China Trends Parasitol 2010 6 264 270 10.1016/j.pt.2010.02.007 20400374

[B10] Wang XB Zhang LX Luo RF Wang GF Chen YD Medina A Eggleston K Rozelle S Smith DS Soil-transmitted helminth infections and correlated risk factors in preschool and school-aged children in rural southwest China PLoS One 2012 6 e45939 10.1371/journal.pone.0045939 23029330 PMC3459941

[B11] Zhou XN Bergquist R Tanner M Elimination of tropical disease through surveillance and response Infect Dis Poverty 2013 6 1 10.1186/2049-9957-2-1 23849433 PMC3707090

[B12] Raso G Vounatsou P Gosoniu L Tanner M N’Goran EK Utzinger J Risk factors and spatial patterns of hookworm infection among schoolchildren in a rural area of western Côte d’Ivoire Int J Parasitol 2006 6 201 210 10.1016/j.ijpara.2005.09.003 16259987

[B13] Pullan RL Gething PW Smith JL Mwandawiro CS Sturrock HJW Gitonga CW Hay SI Brooker S Spatial modelling of soil-transmitted helminth infections in Kenya: a disease control planning tool PLoS Negl Trop Dis 2011 6 e958 10.1371/journal.pntd.0000958 21347451 PMC3035671

[B14] Pullan RL Bethony JM Geiger SM Cundill B Correa-Oliveira R Quinnell RJ Brooker S Human helminth co-infection: analysis of spatial patterns and risk factors in a Brazilian community PLoS Negl Trop Dis 2008 6 e352 10.1371/journal.pntd.0000352 19104658 PMC2602736

[B15] Chammartin F Scholte RGC Guimarães LH Tanner M Utzinger J Vounatsou P Soil-transmitted helminth infection in South America: a systematic review and geostatistical meta-analysis Lancet Infect Dis 2013 6 507 518 10.1016/S1473-3099(13)70071-9 23562238

[B16] Scholte RGC Schur N Bavia ME Carvalho EM Chammartin F Utzinger J Vounatsou P Spatial analysis and risk mapping of soil-transmitted helminth infections in Brazil, using Bayesian geostatiscal models Geosopat Health 2013 6 97 110 10.4081/gh.2013.58 24258887

[B17] Gelfand AE Hills SE Racinepoon A Smith AFM Illustration of Bayesian-inference in normal data models using Gibbs sampling J Am Statist Assoc 1990 6 972 985 10.1080/01621459.1990.10474968

[B18] Diggle PJ Tawn JA Moyeed RA Model-based geostatistics J R Stat Soc Ser C: Appl Stat 1998 6 299 326

[B19] Rue H Martino S Chopin N Approximate Bayesian inference for latent Gaussian models by using integrated nested Laplace approximations J R Stat Soc Ser B: Stat Methodol 2009 6 319 392 10.1111/j.1467-9868.2008.00700.x

[B20] Cameletti M Lindgren F Simpson D Rue H Spatio-temporal modeling of particulate matter concentration through the SPDE approach Adv Stat Anal 2013 6 109 131 10.1007/s10182-012-0196-3

[B21] Hürlimann E Schur N Boutsika K Stensgaard AS Laserna de Himpsl M Ziegelbauer K Laizer N Camenzind L Di Pasquale A Ekpo UF Toward an open-access global database for mapping, control, and surveillance of neglected tropical diseases PLoS Negl Trop Dis 2011 6 e1404 10.1371/journal.pntd.0001404 22180793 PMC3236728

[B22] Sanderson EW Jaiteh M Levy MA Redford KH Wannebo AV Woolmer G The human footprint and the last of the wild Bioscience 2002 6 891 904 10.1641/0006-3568(2002)052[0891:THFATL]2.0.CO;2

[B23] Dormann CF Elith J Bacher S Buchmann C Carl G Carre G Marquez JRG Gruber B Lafourcade B Leitao PJ Collinearity: a review of methods to deal with it and a simulation study evaluating their performance Ecography 2013 6 27 46 10.1111/j.1600-0587.2012.07348.x

[B24] Karagiannis-Voules DA Scholte RGC Guimarães LH Utzinger J Vounatsou P Bayesian geostatistical modeling of leishmaniasis incidence in Brazil PLoS Negl Trop Dis 2013 6 e2213 10.1371/journal.pntd.0002213 23675545 PMC3649962

[B25] Lindgren F Rue H Lindstrom J An explicit link between Gaussian fields and Gaussian Markov random fields: the stochastic partial differential equation approach J R Stat Soc Ser B: Stat Methodol 2011 6 423 498 10.1111/j.1467-9868.2011.00777.x

[B26] Scheipl F Fahrmeir L Kneib T Spike-and-slab priors for function selection in structured additive regression models J Am Statist 2012 6 1518 1532 10.1080/01621459.2012.737742

[B27] Chammartin F Hürlimann E Raso J N’Goran EK Utzinger J Vounatsou P Statistical methodological issues in mapping historical schistosomiasis survey data Acta Trop 2013 6 345 352 10.1016/j.actatropica.2013.04.012 23648217

[B28] Lunn D Spiegelhalter D Thomas A Best N The BUGS project: evolution, critique and future directions Stat Med 2009 6 3049 3067 10.1002/sim.3680 19630097

[B29] Gelman A Rubin DB Inference from iterative simulation using multiple sequences Stat Sci 1992 6 457 511 10.1214/ss/1177011136

[B30] Plummer M Best N Cowles K Vines K CODA: convergence diagnosis and output analysis for MCMC R News 2006 6 7 11

[B31] WHO Prevention and control of schistosomiasis and soil-transmitted helminthiasis: report of a WHO expert committee WHO Tech Rep Ser 2002 6 1 57 12592987

[B32] Keiser J Utzinger J Efficacy of current drugs against soil-transmitted helminth infections: systematic review and meta-analysis JAMA 2008 6 1937 1948 18430913 10.1001/jama.299.16.1937

[B33] Booth M Bundy DAP Comparative prevalences of *Ascaris lumbricoides*, *Trichuris trichiura* and hookworm infections and the prospects for combined control Parasitology 1992 6 151 157 10.1017/S0031182000073807 1437273

[B34] Tchuem Tchuenté LA Behnke JM Gilbert FS Southgate VR Vercruysse J Polyparasitism with *Schistosoma haematobium* and soil-transmitted helminth infections among school children in Loum, Cameroon Trop Med Int Health 2003 6 975 986 10.1046/j.1360-2276.2003.01120.x 14629763

[B35] Tchuem Tchuenté LA Control of soil-transmitted helminths in sub-Saharan Africa: diagnosis, drug efficacy concerns and challenges Acta Trop 2011 6 S4 S11 20654570 10.1016/j.actatropica.2010.07.001

[B36] Flores A Esteban JG Angles R Mas-Coma S Soil-transmitted helminth infections at very high altitude in Bolivia Trans R Soc Trop Med Hyg 2001 6 272 277 10.1016/S0035-9203(01)90232-9 11490995

[B37] Gunawardena K Kumarendran B Ebenezer R Gunasingha MS Pathmeswaran A de Silva N Soil-transmitted helminth infections among plantation sector schoolchildren in Sri Lanka: prevalence after ten years of preventive chemotherapy PLoS Negl Trop Dis 2011 6 e1341 10.1371/journal.pntd.0001341 21980549 PMC3181244

[B38] Zhai S Sun A On the relationship between altitude and economy–the inspiration of altitude effects to the economic development of the Qinghai-Tibet plateau region Nationalities Res Qinghai 2012 6 152 159 (in Chinese)

[B39] Schratz A Pineda MF Reforma LG Fox NM Le AT Tommaso Cavalli-Sforza L Henderson MK Mendoza R Utzinger J Ehrenberg JP Neglected diseases and ethnic minorities in the Western Pacific Region: exploring the links Adv Parasitol 2010 6 79 107 20624529 10.1016/S0065-308X(10)72004-2

[B40] Yap P Du ZW Wu FW Jiang JY Chen R Zhou XN Hattendorf J Utzinger J Steinmann P Rapid re-infection with soil-transmitted helminths after triple-dose albendazole treatment of school-aged children in Yunnan, People’s Republic of China Am J Trop Med Hyg 2013 6 23 31 10.4269/ajtmh.13-0009 23690551 PMC3748482

[B41] Brooker S Clements ACA Bundy DAP Global epidemiology, ecology and control of soil-transmitted helminth infections Adv Parasitol 2006 6 221 261 16647972 10.1016/S0065-308X(05)62007-6 PMC1976253

[B42] Norhayati M Oothuman P Fatmah MS Some risk factors of *Ascaris* and *Trichuris* infection in Malaysian aborigine (Orang Asli) children Med J Malaysia 1998 6 401 407 10971984

[B43] Hohmann H Panzer S Phimpachan C Southivong C Schelp FP Relationship of intestinal parasites to the environment and to behavioral factors in children in the Bolikhamxay province of Lao PDR Southeast Asian J Trop Med Public Health 2001 6 4 13 11485093

[B44] Escobedo AA Canete R Nunez FA Prevalence, risk factors and clinical features associated with intestinal parasitic infections in children from San Juan y Martínez, Pinar del Río, Cuba West Indian Med J 2008 6 377 382 19566020

[B45] Knopp S Mohammed KA Stothard JR Khamis IS Rollinson D Marti H Utzinger J Patterns and risk factors of helminthiasis and anemia in a rural and a peri-urban community in Zanzibar, in the context of helminth control programs PLoS Negl Trop Dis 2010 6 e681 10.1371/journal.pntd.0000681 20485491 PMC2867941

[B46] Pinheiro ID de Castro MF Mitterofhe A Pires FAC Abramo C Ribeiro LC Tibirica SHC Coimbra ES Prevalence and risk factors for giardiasis and soil-transmitted helminthiasis in three municipalities of Southeastern Minas Gerais State, Brazil: risk factors for giardiasis and soil-transmitted helminthiasis Parasitol Res 2011 6 1123 1130 10.1007/s00436-010-2154-x 21243507

[B47] Bergquist R Whittaker M Control of neglected tropical diseases in Asia Pacific: implications for health information priorities Infect Dis Poverty 2012 6 3 10.1186/2049-9957-1-3 23849136 PMC3710194

[B48] Ministry of Health Notice of the Ministry of Public Health concerning publishing “National Control Program on Important Parasitic Diseases in 2006-2015” Gazette of the Ministry of Health of People’s Republic of Chin 2006 6 41 44 (in Chinese)

[B49] Katz N Chaves A Pellegrino J A simple device for quantitative stool thick-smear technique in schistosomiasis mansoni Rev Inst Med Trop S ã o Paulo 1972 6 397 400 4675644

[B50] Speich B Knopp S Mohammed KA Khamis IS Rinaldi L Cringoli G Rollinson D Utzinger J Comparative cost assessment of the Kato-Katz and FLOTAC techniques for soil-transmitted helminth diagnosis in epidemiological surveys Parasit Vectors 2010 6 71 10.1186/1756-3305-3-71 20707931 PMC2936391

[B51] Booth M Vounatsou P N’Goran EK Tanner M Utzinger J The influence of sampling effort and the performance of the Kato-Katz technique in diagnosing *Schistosoma mansoni* and hookworm co-infections in rural Côte d’Ivoire Parasitology 2003 6 525 531 10.1017/S0031182003004128 14700188

