# Supplementary figures and images for: Bayesian geostatistical modelling of soil-transmitted helminth survey data in the People’s Republic of China
Source: Parasit Vectors. 2013 Dec 18;6:359. doi: 10.1186/1756-3305-6-359 (PMC3892068; doi:10.1186/1756-3305-6-359)

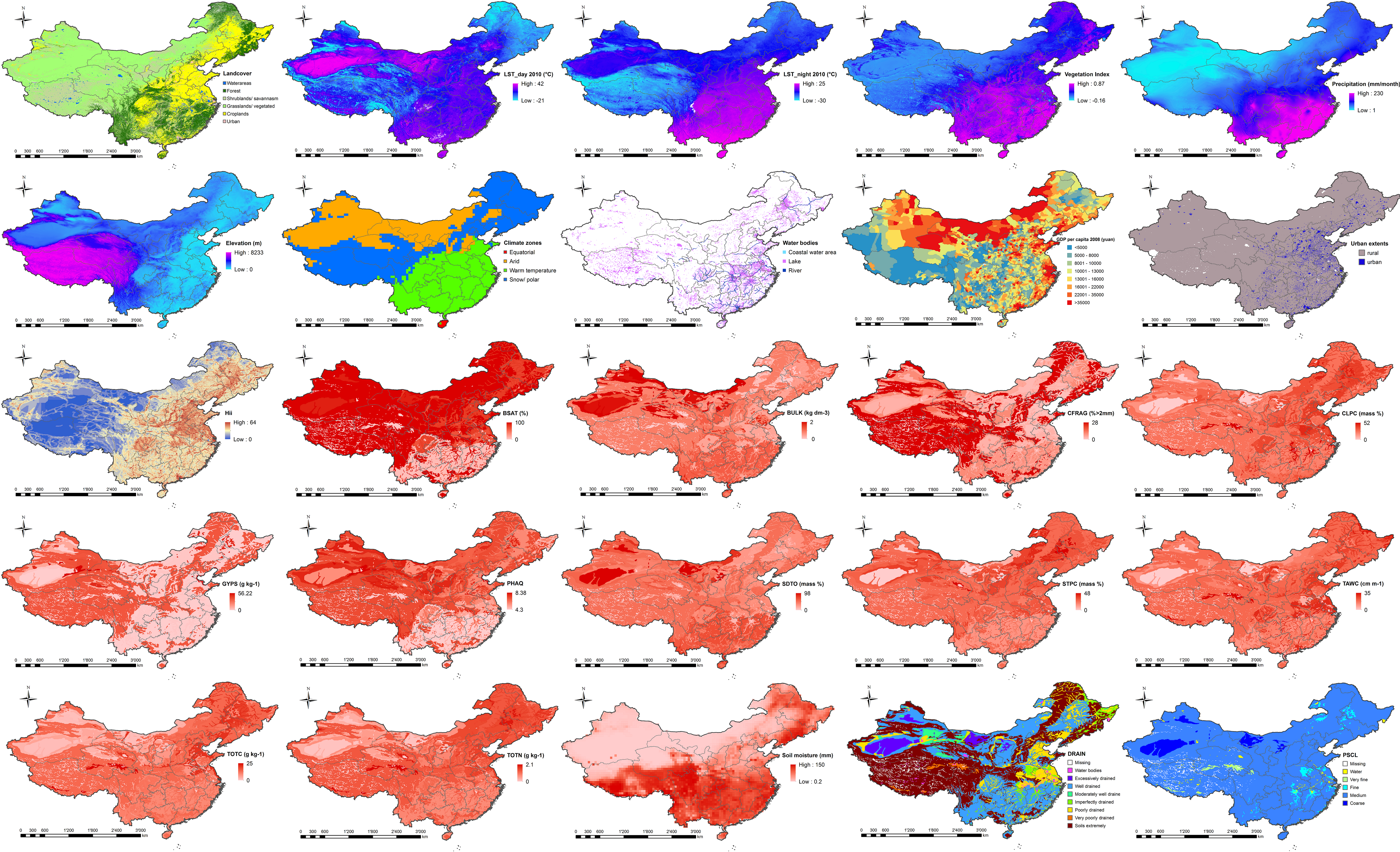

Supplement: Additional file 1: Figure S1 — Spatial distribution of environmental/climatic, soil types and socioeconomic factors in P.R. China. [file 1756-3305-6-359-S1.tiff]

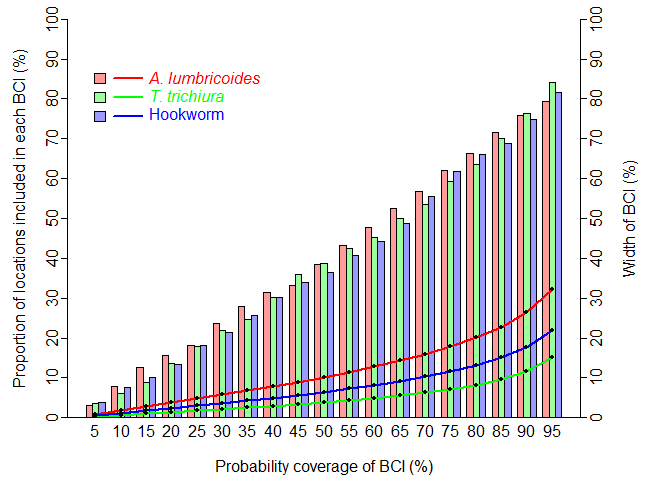

Supplement: Additional file 2: Figure S2 — Model validation results. Percentage of survey locations with observed prevalence included within the Bayesian credible interval (BCI) of various probability coverage cut-offs (bar plots) calculated from the posterior predicted distribution. Solid lines indicate the corresponding width of BCI. [file 1756-3305-6-359-S2.tiff]
